# A non-socially-sensitive predictive model of prostate cancer for Asian males with benign prostatic hyperplasia: A multi-site cross-sectional case-control study

**DOI:** 10.1371/journal.pone.0295608

**Published:** 2023-12-11

**Authors:** Heng-Jui Chang, Sin-Hua Moi, Yu-Jiun Chan, Tzuo-Yun Lan

**Affiliations:** 1 Institute of Public Health, National Yang Ming Chiao Tung University, Taipei, Taiwan; 2 Department of Radiation Oncology, Wesing Surgery Hospital, Kaohsiung, Taiwan; 3 Graduate Institute of Clinical Medicine, College of Medicine, Kaohsiung Medical University, Kaohsiung, Taiwan; 4 Research Center for Precision Environmental Medicine, Kaohsiung Medical University, Kaohsiung, Taiwan; 5 Division of Microbiology, Department of Pathology and Laboratory Medicine, Taipei Veterans General Hospital, Taipei, Taiwan; 6 Center for Infection Control, Taipei Veterans General Hospital, Taipei, Taiwan; 7 Institute of Hospital and Healthcare Administration, National Yang Ming Chiao Tung University, Taipei, Taiwan; Jan Biziel Memorial University Hospital No. 2, POLAND

## Abstract

**Background:**

Benign prostatic hyperplasia (BPH) is common in aging Asian males and is associated with an excess risk of developing prostate cancer (PCa). However, discussions about socially-sensitive experiences such as sexual activity, which can significantly predict PCa risk, may be considered stigmatized in Asian culture. This study aimed to develop a predictive model for PCa risk in Asian males with BPH using non-socially-sensitive information.

**Methods:**

A cross-sectional case-control study, with PCa patients as the cases and remaining as the controls, was conducted on a cohort of Taiwanese males with BPH from four medical institutions. Patients who met the inclusion criteria were enrolled, excluding those aged over 86 years or who had received human papillomavirus (HPV) vaccination. Non-socially-sensitive variables such as obesity, occupational exposure, HPV infection, and PCa family history score (FH score) were included in a fully adjusted logistic regression model, and depicted using a nomogram.

**Results:**

Among 236 BPH patients, 45.3% had PCa. Obesity, occupational exposure, HPV infection, and family history of PCa were significantly associated with PCa risk. The FH score (OR = 1.89, 95% CI = 1.03–3.47, *P* = 0.041) had the highest impact, followed by HPV infection (OR = 1.47, 95% CI = 1.03–2.11, *P* = 0.034), occupational exposure (OR = 1.32, 95% CI = 1.15–1.51, *P* <0.001), and obesity (OR = 1.22, 95% CI = 1.07–1.41, *P* = 0.005). The nomogram accurately depicted the predictive risk, and the model demonstrated robust performance compared to individual factors. In addition, the subgroup analysis results showed elderly age group could obtain more favorable predictive performance in our proposed model (AUC = 0.712).

**Conclusion:**

This non-socially-sensitive predictive model for PCa risk in Taiwanese males with BPH integrates multiple factors that could provide acceptable PCa risk-predictive performance, especially for elderly BPH patients over 70 years, aiding clinical decision-making and early cancer detection.

## Introduction

Benign prostatic hyperplasia (BPH) affects around 70% of aging men, especially prevalent in those aged over 55 years, which is typically caused by cellular hyperplasia [1]. Previous studies reported that men with BPH may relative to excess risk of developing prostate cancer (PCa) compared to the general population [2, 3]. In Asian males, those with BPH may have an increased risk for PCa compared to non-BPH males, especially those with genetic susceptibility [4–6]. Genetic factors are associated with a significant proportion of PCa, with germline mutations reported in approximately 8–12% of cases [7, 8]. This has led to increased discussion regarding genomic evaluations for PCa [9]. PCa exhibits familial aggregation, which sometimes indicating the involvement of inherited or genetic factors [10–12]. Men having a first-degree relative (FDR) or multiple affected relatives diagnosed with PCa are at an increased risk of developing the disease [13]. Previous studies have also revealed that family history in males with BPH was also related to the increased risk of developing PCa [14, 15].

PCa is a complex disease, and while some cases arise sporadically without any family history, many factors may contribute to its development [16, 17]. The human papillomavirus (HPV) may also have a causal role in PCa development [18]. Previous studies have investigated the relationship between HPV infection and PCa risk, and revealed that high risk HPV such as HPV-16 infection was associated with increased risk in PCa development [19]. Obesity is thought to be another possible risk factor, with some studies showing that obese men may have a higher risk of developing more aggressive PCa [16]. Exposure to certain chemicals, such as those experienced by firefighters, may also increase the risk of PCa [16, 20]. Additionally, there is several suggestive evidence discussing the association between exposure to defoliant chemical, named agent orange, and the risk of developing PCa [21]. It has been looked into that there might be a link between sexually transmitted diseases and PCa [22], and our earlier research also worked on whether certain sexual behaviors might influence PCa risk [23].

The non-socially-sensitive information, such as a history of obesity, exposure to chemicals, family history, and HPV infection, could be used to potentially predict the risk of developing PCa in the BPH population. This is particularly crucial in Asian cultures, where discussing sensitive topics like sexual activity may be considered taboo or stigmatized [24]. BPH is a common condition among aging males, and there is a need for better risk prediction tools to help guide clinical decision-making regarding PCa screening. Furthermore, understanding the relationship between these non-socially-sensitive risk factors and PCa risk in Asian males with BPH can help identify high-risk individuals who may benefit from targeted screening such as prostate-specific antigen (PSA) test. Therefore, this study is designed to develop a predictive model for PCa risk in Asian males with BPH using non-socially-sensitive information.

## Materials and methods

### Ethics statement

Institutional review board (IRB) approvals were obtained from Min Sheng General Hospital (MSIRB No. 2017012), Landseed International Hospital (LSIHIRB No. 18-041-A2), Taoyuan General Hospital (IRB No. TVGH109004) and Ten Chen Hospital (TCH IRB No. 109-A-05-01). Informed consent was obtained from all individual participants included in the study.

### Data source

This is a cross-sectional case-control study conducted on a cohort of Asian males with BPH from four medical institutions under approved protocol with the principles of the Declaration of Helsinki. The inclusion criteria for this study encompassed males who were pathologically diagnosed with BPH, exhibited symptoms, had a history of drug use, and regularly visited the outpatient department (OPD), but had no previous PCa diagnosis. BPH patients who had visited the OPD between February 2018 and December 2020 were enrolled in the study after providing informed consent. Individuals aged over 86 years or those who had previously received HPV vaccination were excluded. A total of 236 BPH patients were enrolled. The sample size calculation for study cohort have been well described in our previous work [23].

### Prostate cancer diagnosis

Prostate cancer diagnoses were conducted for all enrolled BPH patients during the study period. Consequently, the exact date of the initial BPH diagnosis for the study cohort could not be determined. All BPH patients underwent a prostate cancer diagnosis upon enrollment in this study. PCa cases included those with pathological confirmation of PCa, aged between 55 and 86 years. Most of these cases had biopsies due to elevated PSA levels and suspicion of PCa. A smaller number of cases were incidentally discovered to have malignant PCa during transurethral resection of the prostate (TURP) for BPH symptoms. Controls, or confirmed non-PCa cases, were defined as BPH patients with clinically apparent lower urinary tract symptoms (LUTS) who were refractory to oral medications, required urethral catheterization, and underwent TURP procedures by urologists. BPH patients with incidental PSA elevation who were highly suspected of having PCa but had no cancer cells detected in their TRUS biopsies were also categorized as controls. The primary surgical procedures included radical prostatectomy (RP), transurethral resection of the prostate (TURP), and transrectal ultrasound (TRUS) biopsy, with RP being exclusive to the PCa group. In summary, the PCa risk estimated in this study represents a cross-sectional or time-point risk rather than a lifetime PCa risk. Overall, 107 (45.3%) of BPH patients were diagnosed with PCa during the study, with the remaining 129 patients serving as controls.

### Non-socially-sensitive factors

We collected non-socially-sensitive factors using a validated questionnaire administered by qualified interviewers in-person. The variables collected via questionnaire included body mass index (BMI), occupational exposure, and family history PCa in the study cohort. Patients with a BMI over 27 were classified as obese according to the health promotion administration (HPA) guidelines. Patients who had ever been exposed to chemicals or substances associated with the development of PCa in occupational settings were considered exposed. The investigated exposure factors included plasticizer, chemicals powder or solution, and radiation. We investigated family members who had been affected by PCa in the study cohort and transformed this information into a family history severity score, which is introduced at the following section. HPV infection was evaluated using formalin-fixed, paraffin-embedded tissue (FFPE) samples at the Taipei Institute of Pathology. Informed consents were obtained from the study population prior to sample collection. Each FFPE sample was tested using two commercial kits: Cobas 4800 HPV Test (Roche Molecular Systems) and DR HPV Genotyping IVD Kit (DR. Chip Biotech).

### Family history severity score (FH score)

The relationship of the family member and the number affected by PCa was recorded, and we further classified the family members based on their relationship into FDR, second-degree relatives (SDR), and third-degree relatives (TDR). FDRs comprised the father and son of the study cohort, SDRs the grandfather, brother, and grandson, and TDRs the granduncle, uncle, and cousin. The FH severity is the weighted sum of relatives who have PCa, with each FDR, SDR, and TDR contributing 0.5, 0.25, and 0.125, respectively. The FH score was the summation of the FH severity according to the family history of the study cohort, which could be computed using the following Eq (1):

$$FH\;score=\sum {\left(n\times s\right)}_{d}$$ (1)

where *n* denotes the number of affected family members, *s* denotes the FH severity, and *d* denotes degree of relative, including FDR, SDR, and TDR.

### Statistical analysis

The characteristics of the study cohort were summarized using median and range for continuous variables, while frequency and percentage were used for categorical variables. Differences in characteristics between the PCa and control groups were estimated using Mann-Whitney, chi-squared, and Fisher’s exact tests. Non-socially-sensitive variables, including obesity, occupational exposure, HPV infection, and FH score, were included in the multivariate fully adjusted binomial logistic regression model to evaluate the impact of corresponding factorson PCa risk. A nomogram was used to illustrate the PCa risk prediction of BPH patients according to the proposed model. In addition, subgroup analyses for younger and elder subgroup divided by the median age of study cohort were performed. Furthermore, the predictive performance of the proposed model was recalibrated by estimating the predicted probability versus actual probability for PCa risk. The comparison of predictive performance between the proposed model and single non-socially-sensitive factors was estimated using receiver operating characteristics (ROC) tests. The C-index for the proposed model and each single factor were computed, with higher C-index values indicating better predictive performance. All *p*-values were two-sided, and a *p*-value less than 0.05 was considered statistically significant. All analyses were performed using R software version 4.3.0 (R Core team, Vienna, Austria, 2023).

## Results

The characteristics of study cohort was summarized in Table 1. The median enrollment age in the study cohort was 70.9 years, ranging from 55.3 to 85.9. The median age of both the PCa and control groups were similar, at 70.5 (range: 55.3 to 85.9) and 71.6 (range: 55.4 to 85.7) years, respectively. The BMI of the study cohort ranged from 14.2 to 34.7, with an overall 25.4% (n = 60) of patients classified as obese. Among the study population, 28.8% (n = 68) reported exposure to occupational risks, while only 3.0% (n = 7) were infected with HPV. PCa patients exhibited a significantly higher proportion of obesity (33.6% vs 18.6% in controls, *P* = 0.008) and occupational exposure (41.1% vs 18.6% in controls, *P* < 0.001), but not HPV infection (4.7% vs 1.6% in controls, *P* = 0.250). In addition, three controls have previously been diagnosed as other cancers, one with colon cancer, and two with lung cancer. While no other cancers or severe diseases were found in PCa patients.

**Table 1:** Characteristics of study cohort.

Characteristics	Overall (n = 236)	Controls (n = 129)	PCa (n = 107)	*P*
Age	70.9 (55.3–85.9)	71.6 (55.4–85.7)	70.5 (55.3–85.9)	0.854
BMI	24.6 (14.2–34.7)	23.7 (14.2–33.1)	25.9 (19.9–34.7)	**<0.001**
Obesity (BMI>27)	60 (25.4%)	24 (18.6%)	36 (33.6%)	**0.008**
Occupational exposure	68 (28.8%)	24 (18.6%)	44 (41.1%)	**<0.001**
HPV infection	7 (3.0%)	2 (1.6%)	5 (4.7%)	0.250
Family history				
Any PCa	13 (5.5%)	3 (2.3%)	10 (9.3%)	**0.019**
FDR	2 (0.8%)	0 (0.0%)	2 (1.9%)	0.205
SDR	11 (4.7%)	3 (2.3%)	8 (7.5%)	0.071
TDR	3 (1.3%)	1 (0.8%)	2 (1.9%)	0.592
FH Score				**0.040**
0	223 (94.5%)	126 (97.7%)	97 (90.7%)	
0.125	2 (0.8%)	0 (0.0%)	2 (1.9%)	
0.25	6 (2.5%)	2 (1.6%)	4 (3.7%)	
0.5	2 (0.8%)	0 (0.0%)	2 (1.9%)	
0.625	1 (0.4%)	1 (0.8%)	0 (0.0%)	
0.75	2 (0.8%)	0 (0.0%)	2 (1.9%)	

PCa, prostate cancer. BMI, body mass index. HPV, human papillomavirus. FDR, first-degree relative. SDR, second-degree relative. TDR, third-degree relative. FH score, Family history severity score.

A total of 13 (5.5%) patients reported a family history of PCa, with the proportion being significantly higher in the PCa group compared to controls (9.3% vs 2.3%, *P* < 0.001). Although the distribution of the degree of relationship including FDR, SDR, and TDR between the two groups was not significantly different, the FH score showed a significant difference between the PCa and control groups (*P* = 0.040), with the former exhibiting a higher score. Specifically, 2 (1.9%), 2 (1.9%), 4 (3.7%), and 2 (1.9%) PCa patients were scored 0.75, 0.5, 0.25, and 0.125, respectively, while only 1 (0.8%) and 2 (1.6%) controls were scored 0.625 and 0.25, respectively.

Table 2 presented the results of the multivariate logistic regression analysis. All retained non-socially-sensitive factors were found to be significantly associated with the risk of developing PCa. The FH score (OR = 1.89, 95% CI = 1.03–3.47, *P* = 0.041) had the highest impact on the risk, followed by HPV infection (OR = 1.47, 95% CI = 1.03–2.11, *P* = 0.034), occupational exposure (OR = 1.32, 95% CI = 1.15–1.51, *P* <0.001), and obesity (OR = 1.22, 95% CI = 1.07–1.41, *P* = 0.005).

**Table 2:** Multivariate logistic regression model for PCa risk.

Characteristics	Comparison	Coefficients	OR (95% CI)	*P*
Obesity	Obesity vs Normal	0.202	1.22 (1.07, 1.41)	0.005
Occupational exposure	Exposed vs None	0.277	1.32 (1.15, 1.51)	<0.001
HPV infection	Positive vs Negative	0.388	1.47 (1.03, 2.11)	0.034
FH Score	0~0.75	0.637	1.89 (1.03, 3.47)	0.041

PCa, prostate cancer. HPV, human papillomavirus. FH score, Family history severity score.

**Fig 1:**
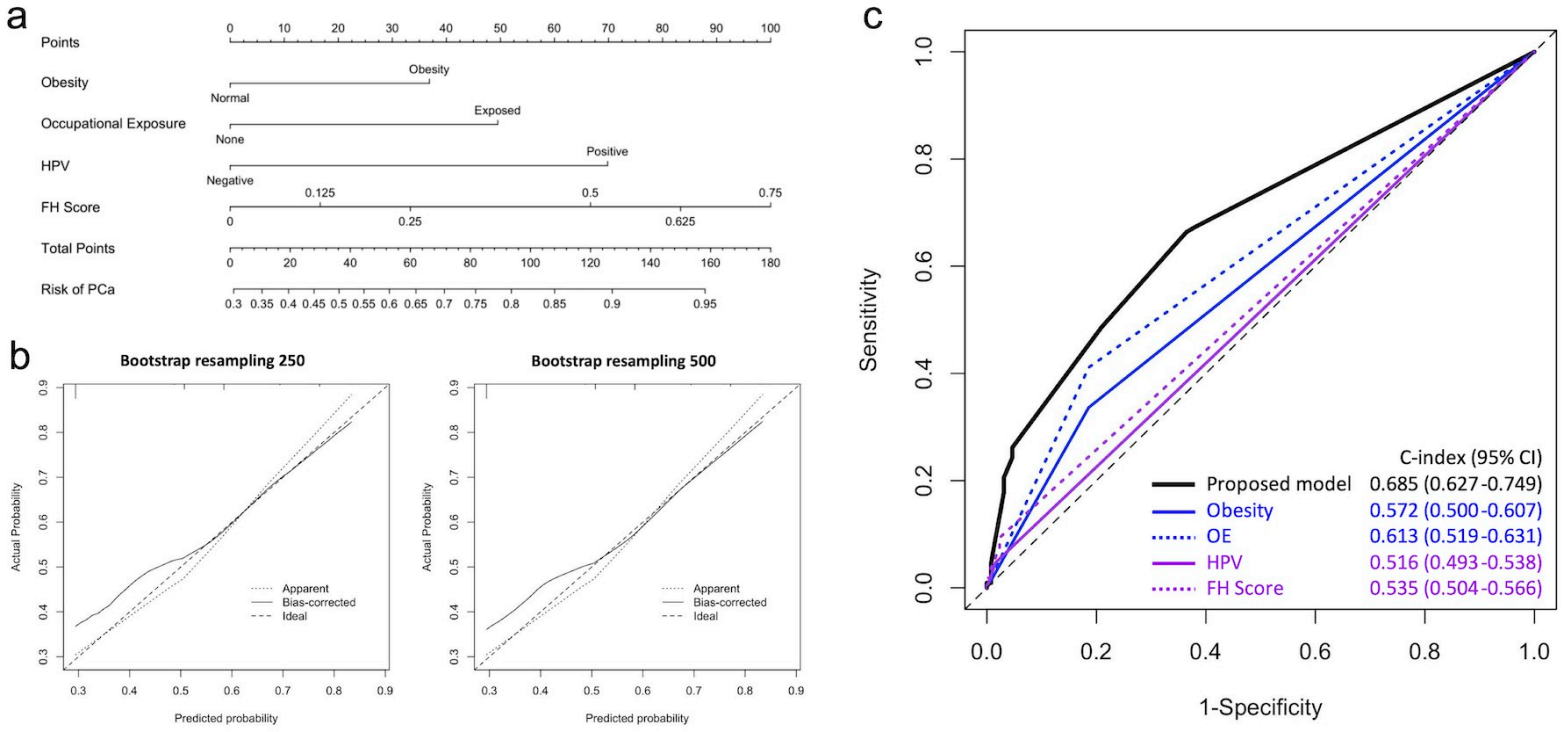
The predictive performance of proposed model for PCa risk in all patients. (a) Nomogram for PCa risk prediction of benign prostatic hyperplasia patients. (b) The predicted probability versus actual probability of proposed model for PCa with 250 and 500 resampling using bootstrap method. (c) Comparison results of predictive performance between proposed model and single risk factors of PCa. PCa, prostate cancer. OE, Occupational exposure. HPV, human papillomavirus. FH score, Family history severity score.

The nomogram in Fig 1a illustrated the predictive risk of each non-socially-sensitive factor, which was consistent with the multivariate model results. The performance of the proposed model containing these four factors was recalibrated using 250-and 500-resampling bootstrap methods, and the predicted and actual probabilities were estimated, as shown in Fig 1b. The recalibration results revealed that both the apparent and bias-corrected probability of the proposed model were close to the ideal line, indicating robust performance for PCa risk prediction. Furthermore, the predictive performance of the proposed model and each single non-socially-sensitive factor were compared using ROC plots, as shown in Fig 1c. The results showed that the proposed model (C-index = 0.685, 95% CI = 0.627–0.749) provided more satisfactory predictive results compared to single factors. Occupational exposure had a C-index of 0.613, followed by obesity, FH score, and HPV infection, with C-indices of 0.572, 0.535, and 0.516, respectively. The unsatisfactory predictive performance of FH score might mainly due to the low specificity results. Despite of that, the proposed model still provides a novel approach for PCa risk prediction by combining these non-socially-sensitive factors.

**Fig 2:**
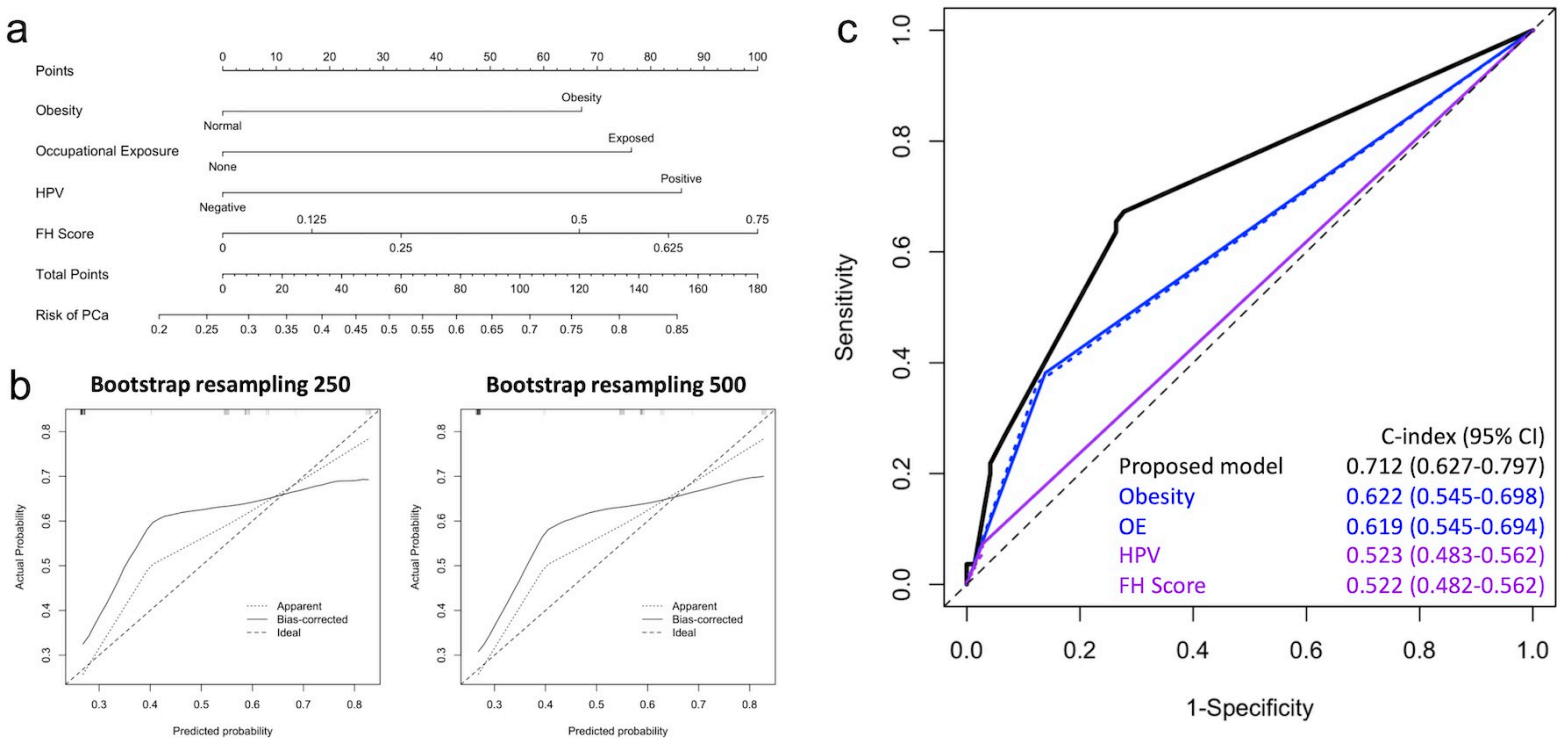
Subgroup analysis results for elder patients aged over 70 years (n = 127). (a) Nomogram for PCa risk prediction of benign prostatic hyperplasia patients. (b) The predicted probability versus actual probability of the proposed model for PCa with 250 and 500 resampling using the bootstrap method. (c) Comparison results of predictive performance between the proposed model and single risk factors of PCa. PCa, prostate cancer. OE, Occupational exposure. HPV, human papillomavirus. FH score, Family history severity score.

Since aging is pivotal for PCa risk development, we further established a subgroup analysis for younger and elder groups. All patients were divided into younger (≤70 years) and elder (>70 years) age subgroups according to the median age (70.9 years) of the study cohort. 127 patients were classified as the elder subgroup, and 109 were divided into the younger age subgroup. 52 (47.7%) patients in the younger subgroup have been diagnosed with PCa, and 55 (43.3%) patients in the elder subgroup have been diagnosed with PCa. The subgroup analysis results for elder patients aged over 70 years were summarized in Fig 2. The subgroup analysis results showed elderly age group could obtain more favorable predictive performance in our proposed model (AUC = 0.712), as shown in Fig 2c. While comparing the nomogram between all patients (Fig 1a) and the elder subgroup (Fig 2b), we noticed that obesity and occupational exposure could have a greater impact on PCa risk development in the elder subgroup. In addition, the subgroup analysis results for younger patients who were aged under 70 years were summarized in Supplementary results (S1 Fig). The younger age subgroup showed less favorable predictive performance than the elder subgroup or all patients. Taken together, our proposed model integrating obesity, FH score, occupational exposure, and HPV infection for PCa risk development could obtain more favorable predictive results for the elder subgroup.

## Discussion

In the best of our knowledge, this is the first study proposed a non-socially-sensitive predictive model for PCa development in Taiwanese males with BPH. The proposed model integrates multiple risk factors, including obesity, family history, occupational exposure, and HPV infection, to more comprehensive risk estimation for PCa development in Asian males with BPH, particularly for elder BPH patients who aged over 70 years.

Based on our findings, there is evidence suggesting that patients characterized by obesity, family history, occupational exposure, and HPV infection may have an elevated risk of developing PCa, which is consistent with previous research findings. Obesity has been identified as a risk factor for various types of cancer, including PCa [25]. The elevation of leptin concentration could potentially stimulate the prostate growth and angiogenesis, and later increased risk of PCa [26]. Studies have found that men with a higher BMI may have an increased risk of developing PCa, and the risk may be even higher in Asian populations with a lower average BMI [27]. Family history is a well-established risk factor for PCa, and previous risk estimation tools such as PCa risk calculator, have also considered family history as an important factor [28]. The risk of PCa can be influenced by various occupational factors, and exposure to certain harmful agents in the workplace has been suggested as a potential contributing factor [29]. In addition, occupational exposures are more common in Asian population which may contribute to the increase risk of PCa [30].

Recent studies have reported that males with HPV-16 infection may increase risk of developing PCa, and the HPV infection has been proposed and considered to play a role in the development of malignant tumor [31]. However, the interactions between these non-socially genetic and environmental factors may need to be explored to provide more in-depth risk profiles for PCa development.

Aging is a well-established risk factor for prostate cancer (PCa). Notably, our subgroup analysis results showed that obesity and occupational exposure could have a greater impact on PCa risk development in the elderly subgroup, while family history could have a higher impact in the younger subgroup. This finding is consistent with previous studies, which demonstrated that aging patients have a lower tolerance for exogenous exposure risks related to PCa [32–34]. Additionally, men with multiple affected relatives, especially if the diagnosis was made at a young age, might have a greater risk of developing PCa [13]. Furthermore, the aging process can increase the risk of HPV infection in males with BPH. In this study, one patient in the younger subgroup was infected by HPV, but six patients in the elder subgroup were HPV infected. Elderly patients may have a weaker immune system, making them more susceptible to HPV, and further heightened the risk for PCa [35, 36].

However, several limitations of current study should be noted. First, the research was conducted on Asian males with BPH, which may limit the generalizability of the findings to different cultures. Second, the non-socially-sensitive information were collected using self-reported questionnaire, which may involve recall bias. Furthermore, HPV infection is not currently standard practice in clinical settings, thus, incorporating HPV examination into routine screening protocols would require additional costs and resources. In addition, the restricted sample size might limit the predictive power results for our study. The exact date of the initial BPH diagnosis for the study cohort could not be determined due to the nature of the case-control study. Therefore, the actual period from the initial BPH diagnosis to PCa development was incomparable. To overcome these limitations and further enhance the validity of the proposed model, future research should aim to include larger sample sizes and more diverse populations.

Currently, PSA testing is regarded as a crucial tool for identifying both BPH and PCa, and there is currently no better tumor marker than PSA. However, it is remaining challenges because PSA test results could be altered due to natural fluctuations in PSA levels over time [37–39]. The fluctuations can make it difficult to consistently and accurately interpret the results, potentially resulting in the underestimation of PCa [40]. When it comes to clinical practice in Taiwan, PSA screening is usually not widely adopted, and so PSA tests are more likely performed to symptomatic males, which can result in the detection of PCa at more advanced stages and higher mortality rate. By monitoring changes in PSA levels over time (PSA velocity) and considering the ratio of PSA to prostate volume (PSA density) can provide additional information for risk assessment [41–43]. In general, rapid increases in PSA levels or high PSA density may indicate a higher risk of aggressive PCa [42, 44]. Therefore, the proposed risk predictive model could contribute as guidance for implementing PSA testing earlier or for follow-up, in order to improve early detection of PCa and avoid delayed treatment.

The observed variables in our proposed model are not sensitive information which could let the researcher reaching more accurate information and reduce the information bias due to the sensitive issue such as sexual experience. This study is particularly contributing for clinical decision-making and early cancer detection for Asian males with more sexually conservative cultures. Additionally, it would be valuable to conduct a cost-effectiveness analysis to assess the practicality and economic implications of implementing the proposed model for PCa screening in Asian males with BPH. Understanding the potential benefits and drawbacks in terms of healthcare resource utilization and patient outcomes can guide decision-making and inform healthcare policies regarding screening strategies.

## Conclusion

Our study demonstrated that the proposed model, integrating obesity, family history, occupational exposure, and HPV infection, could provide acceptable PCa risk-predictive performance for elderly BPH patients aged over 70 years. This non-socially-sensitive case-control study is important because it addresses a significant gap in the literature and provides a better understanding of the relationship between non-socially-sensitive risk factors and PCa risk in Asian males with BPH. In summary, the proposed non-socially-sensitive predictive model for PCa could contribute for the early detection and clinical decision-making, especially in Asian males with more sexually conservative cultures. While PSA testing is essential, the fluctuation of PSA levels over time poses challenges in interpretation. Monitoring both PSA velocity and density can provide additional risk assessment information. Implementing an early risk predictive model can remind BPH patients at risk and physicians to conduct regular PSA testing or follow-up, increasing the early detection rate and ensuring timely treatment for PCa.

## Supporting information

S1 Fig Subgroup analysis results for younger patients aged under 70 years (n = 109). (a) Nomogram for PCa risk prediction of benign prostatic hyperplasia patients. (b) The predicted probability versus actual probability of the proposed model for PCa with 250 and 500 resampling using the bootstrap method. (c) Comparison results of predictive performance between the proposed model and single risk factors of PCa. PCa, prostate cancer. OE, Occupational exposure. HPV, human papillomavirus. FH score, Family history severity score.
(TIF)
