# Supplementary figures and images for: A non-socially-sensitive predictive model of prostate cancer for Asian males with benign prostatic hyperplasia: A multi-site cross-sectional case-control study
Source: PLoS One. 2023 Dec 11;18(12):e0295608. doi: 10.1371/journal.pone.0295608 (PMC10712871; doi:10.1371/journal.pone.0295608)

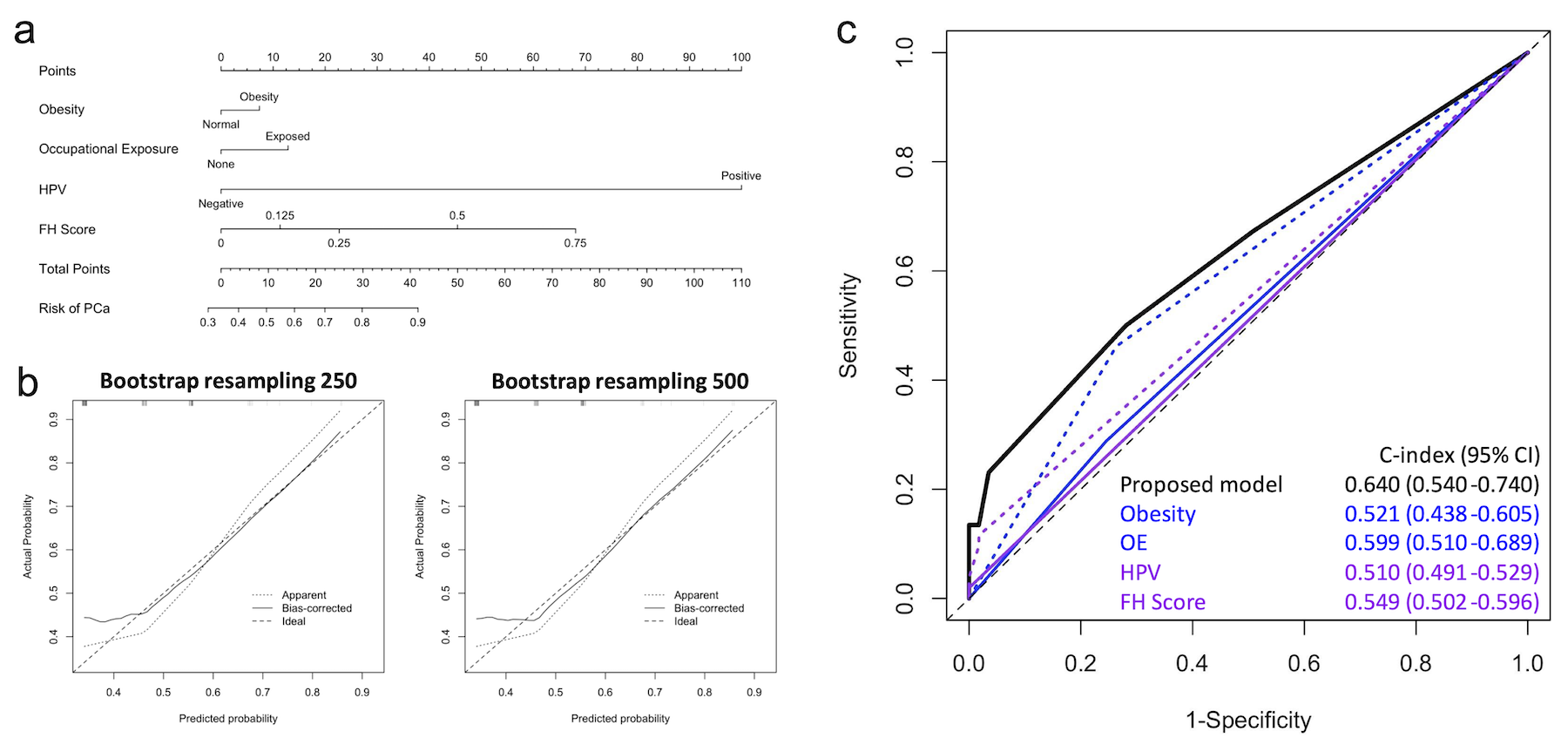

Supplement: S1 Fig — (a) Nomogram for PCa risk prediction of benign prostatic hyperplasia patients. (b) The predicted probability versus actual probability of the proposed model for PCa with 250 and 500 resampling using the bootstrap method. (c) Comparison results of predictive performance between the proposed model and single risk factors of PCa. PCa, prostate cancer. OE, Occupational exposure. HPV, human papillomavirus. FH score, Family history severity score. (TIF) [file pone.0295608.s001.tif]
